# Supplementary material for: Modelling Skylarks (Alauda arvensis) to Predict Impacts of Changes in Land Management and Policy: Development and Testing of an Agent-Based Model
Source: PLoS One. 2013 Jun 6;8(6):e65803. doi: 10.1371/journal.pone.0065803 (PMC3675089; doi:10.1371/journal.pone.0065803)
Supplement: Supporting Information S4 — The skylark ODdox as a zipped archive. (ZIP) [file pone.0065803.s004.zip › Skylark_ODdox/files.html]

ALMaSS Skylark ODdox: File List


ALMaSS Skylark ODdox
 2.0


- Main Page
- Related Pages
- Classes
- Files

- File List
- File Members

File List

Here is a list of all files with brief descriptions:

|  |  |
| --- | --- |
| ALMaSS\_CmdLine.cpp |  |
| ALMaSS\_Setup.h |  |
| BinaryMapBase.cpp |  |
| BinaryMapBase.h |  |
| BoostRandomGenerators.h | **BoostRandomGenerators.h Boost headers for using the boost random number generation utilities** |
| calendar.cpp |  |
| calendar.h |  |
| CIPELandscapeMaker.h |  |
| configurator.cpp |  |
| configurator.h |  |
| croprotation.cpp |  |
| croprotation.h |  |
| daylength.h |  |
| elements.cpp |  |
| elements.h |  |
| farm.cpp | **Farm.cpp This file contains the source for the Farm class** |
| farm.h | **Farm.h This file contains the headers for the Farm class** |
| farmfuncs.cpp | **Farmfuncs.cpp This file contains the source for implementing the farm events** |
| hedgebanks.cpp |  |
| Landscape.cpp |  |
| landscape.h |  |
| lowqueue.h | **lowqueue.h This file contains the headers for the farm event queue classes** |
| ls.h |  |
| map\_cfg.cpp |  |
| map\_cfg.h |  |
| maperrormsg.cpp |  |
| maperrormsg.h |  |
| misc.cpp |  |
| MovementMap.cpp | **Movementmap.cpp This file contains the source for the MovementMap class** |
| MovementMap.h | **Movementmap.h This file contains the headers for the MovementMap class** |
| pesticide.cpp |  |
| pesticide.h |  |
| plants.cpp |  |
| plants.h |  |
| PopulationManager.cpp | **PopulationManager.cpp This is the code file for the population manager and associated classes** |
| populationmanager.h | **PopulationManager.h This is the header file for the population manager and associated classes** |
| PositionMap.cpp | **PositionMap.cpp This file contains the source for the PositionMap class** |
| positionmap.h | **PositionMap.h This file contains the headers for the PositionMap class** |
| rastermap.cpp |  |
| rastermap.h |  |
| References\_ODDox.h |  |
| Rodenticide.cpp |  |
| Rodenticide.h |  |
| SkylarkConfigs.cpp |  |
| skylarks\_all.cpp |  |
| skylarks\_all.h |  |
| tole\_declaration.h |  |
| tov\_declaration.h |  |
| treatment.h |  |
| weather.cpp |  |
| weather.h |  |


- Generated on Thu Jan 10 2013 13:15:39 for ALMaSS Skylark ODdox by
   1.8.1.1
